# Speeches at the Festival Dinner, 1909

**Published:** 1909-12-04

**Authors:** Edgar Speyer

**Affiliations:** CHAIRMAN


### SPEECHES AT THE FESTIVAL DINNER, 1909.

### SIR EDGAR SPEYER, CHAIRMAN.

In giving you the toast of the evening, "Success to the Great Northern Central Hospital," I need only briefly refer to the many claims it has on us. All that can be said in favour of supporting this excellent institution, which is an absolute necessity to Islington—we cannot imagine the North of London without this hospital—has been said and written in terms more eloquent and more convincing than I can command. In trying to collect the urgently needed funds we have exhausted all arguments, all appeals to reason, to the human heart, to the pockets of Londoners, and it would be a stale tale were I to reiterate in detail why all those who know the beneficent work this hospital does, so strongly feel the paramount necessity of enabling the management not to curtail but to increase its ministration for the good of our poorer fellow-men in the North of London. Since I consented to take the chair here to-night I have given some time and consideration to the affairs of this Hospital, and must say that its financial position gives cause for anxiety. I hope the Committee will forgive me if I speak quite frankly, as friends should do, and I hope they will consider me a friend. Let me preface my remarks by saying that I am addressing you not as a member of the King Edward's Hospital Fund but in my private capacity as one keenly interested in charitable, and especially hospital, work.

#### The Present Position.

Let us look at your position now, gentlemen of the Committee. What do we find? Going back to 1903, we find the following annual deficiencies: 1903, £3,700; 1904, £3,000; 1905, £5,400; 1906, £4,600, surplus owing to £9,787 legacies; 1907, £1,100, surplus owing to £4,673 legacies; 1908, £4,600; 1909, £3,000. There is a total deficiency of £12,875, and, including an overdraft on the Building Fund, the total indebtedness to your bankers amounts to £14,462. It seems to me, and I speak as a business man, that this is exceedingly unsatisfactory and alarming. In my humble opinion, the aim and duty of the management should be to get solvent, not only solvent as regards repayment of their debt, but solvent also annually, by cutting the coat according to the cloth. These chronic deficits are demoralising. Business principles, which guide every well-conducted concern, apply with equal force to hospitals, for, after all, the management of a hospital requires the same qualities as that of any commercial enterprise. And one of the first business rules is to pay your bills. Your debts have almost entirely arisen through accumulation of annual deficits, and I venture to think that, having regard to the financial position of the hospital, no time should be lost and no effort should be spared in stopping these annual deficits. Having been on hospital committees myself, I know how difficult it is to make a budget, how difficult it is to gauge the amount of donations, and especially the amount of legacies. It is this latter item which frequently upsets anticipations; and, looking at the figures of your hospital, it seems to me that to a large extent the heavy reduction in legacies has increased your difficulties.

How to Face the Difficulty.

For the year 1909 (I do not wish to weary you with many figures) you are short of about £3,000. How can this shortage be reduced in coming years? There are two ways. One is to do as some other hospitals have done, and make a small charge to out-patients who can afford to pay. I know this is an unpopular proposal, and the medical staff especially think it a mistake, but I cannot but think that, everything considered, it is in the true and permanent interest of the hospital and those that depend on it. You have to increase your income somehow. You will soon have 30,000 out-patients. Supposing they paid 3d. each for medicines and bandages, this would bring you in 90,000 pence, or £375, but it would probably be somewhat less, as a certain proportion will not be able to pay. On the other hand, the 2,000 in-patients, or their relatives and friends, could in many cases be asked to pay something, so that I think you may easily get from these sources £400 per annum, but that of course is not much. Still it helps. My second point is that still stricter economy will have to be practised in all departments. I am glad to be able to state, after looking carefully at your figures, that from the point of view of economy there is little criticism to make. As a matter of fact, the cost per bed occupied during the year 1908 was only £3 8s. 2d. higher than the average cost of the large hospitals, but there is, as you see, a small margin for improvement, and, if you bring down your cost to the average—£81 10s. 9d. per bed—the saving would then be something like £350 per annum. This may be a counsel of perfection, but if I had the management of this hospital these are the lines I would work on. I understand that a house-to-house canvass is made, and that the neighbourhood does subscribe to a certain extent. I am well aware that Islington is a poor neighbourhood, but surely the many shopkeepers and others could be prevailed upon to help their only hospital in the district. The middle classes are too apathetic in these matters. They should realise their duty towards institutions such as these and remember that if they persist in this indifference their money may have to be got finally through the rates, for the sick poor must be taken care of. That is not a question of charity; it is demanded by civilisation and humanity. I cannot but think, if the points I have ventured to make are seriously taken in hand, that the financial and general efficiency of the Hospital will be greatly increased. It is the small things that count, and believe me—I speak from experience—the public will support you more when you are solvent than when you are in debt. We have found this at Poplar, where we never have been in debt although that hospital is situate in one of the poorest districts. I feel very strongly that no hospital should keep its wards open if it cannot pay for them. If you find after having cleaned your slate and having applied the strictest tests as to all possible economies, that with all the help you are able to enlist, including the King Edward's Hospital Fund, you cannot make both ends meet, well then you must close some wards. But I trust it will not come to that sad calamity, for no one realises more than I do what this would mean to your neighbourhood.

A Debt of £14,452.

And now let us see how we stand. Your debt amounts to £14,462. If it had been possible, I would have suggested that the Hospital should sell some of its investments to get clear, but I understand that all your invested funds are endowed, so that is not possible. It is therefore, clear that, taking a most sanguine view of the result of to-night's dinner, you will continue to be in debt, and I can only hope that increased future legacies, donations, and more annual subscriptions will soon put you straight. But I do venture to hope that my suggestion about out-patients' payments will be seriously considered, because in other places it has answered. This is not a novel proposal. I well remember when it was suggested at the London and Poplar hospitals. There was a great deal of opposition at first, but it was overcome and patients' payments once established have answered splendidly. Business methods are not incompatible with sympathy; in fact, they enable transient sympathy to be translated into enduring acts of sympathy:

> "I must be cruel, only to be kind:
> Thus bad begins, and worse remains behind."

Giving indiscriminately is one of the worst forms of pauperising, and as the Royal Commission on the Poor Law in their most instructive chapter on medical assistance point out, there should be a method in these matters, which does not diminish but encourage and stimulate a feeling of independence and self-maintenance. When one reads that Report one cannot but be struck with the great strides that have been made in this country during the last fifty years in caring for the sick and infirm; and therein lies the hope for better things to come. And come they must, for reforms are sadly and urgently needed. If we remember that sickness is one of the chief causes of pauperism, and that the more we can eliminate or diminish sickness among the poor the more we shall eliminate a great part of existing distress, the importance of hospital treatment as a great factor in our social organisation becomes apparent. I sincerely trust that Poor Law reform will be taken in hand by this or the next Government, be it Liberal or Conservative, and with it hospital reform. It is long overdue. Admirable as is the work done by our voluntary hospitals, a thorough overhauling of all the agencies that tend the sick poor is imperative.

Poor Law Infirmaries and Voluntary Hospitals.

A short time ago when visiting this Hospital in order to get acquainted with its work I went to see the nearest infirmary, at Highgate; and, going through its fine wards, I asked myself and asked the resident medical officer, Dr. Robinson, Where is the dividing-line between this Infirmary and this Hospital? We need not specialise, but ask the question generally. As far as I can make out there really is none. The fact is, many of the infirmaries are equal if not superior in bed space and style of buildings to general hospitals. In a number of them the most delicate surgical operations are performed. Three thousand in London infirmaries alone! It is undoubtedly true that the great advance in medical science has driven people into the infirmaries, and they are practically State- or rate-aided hospitals. Voluntary hospitals cannot cope with the increasing demands made on them by modern hygienic requirements, due to the progress of medical knowledge, and are less and less able to find the vast sums of money needed to provide what has now become essential. That pressure is bound to increase. As the Royal Commission points out, there should be systematic—and, what is equally important, sympathetic—co-operation between public assistance authorities, sanitary authorities, and voluntary medical institutions, with a clear definition of their respective functions. It seems hardly credible that such overlapping, such want of system, and consequently such waste have been allowed to exist. And in a smaller way this want of co-operation is apparent amongst voluntary hospitals themselves. What a pity it is to see half a dozen hospitals in a small area keeping sternly apart as if their interests were not identical! In many ways they might combine and produce economies which would enable them to extend their labours. Take the question of keeping stores. As has been pointed out by Mr. Morris, twenty expert chemists and twenty expert stewards all examining the same kind of samples at twenty hospitals at contract time, while one chemist and one steward could do this work if properly organised. My business instinct revolts at such practices. Why not have a central buying authority?

### A Call for Reform.

Ladies and gentlemen, I venture to think that individualism in charity is carried too far in this country. It produces chaos and muddle, and although we may muddle through somehow, that muddle, and especially that "somehow," is an expensive process, expensive in time, in money, in human lives. Let us remember that order and method and system and organisation are labour-saving machines. It seems to me that the time of unsupported, unorganised private charity will soon be behind us. We must get rid of what Mr. Lilly has aptly called the "anarchy of individualism." The problems are too great, too serious to be left only to amateurs, however devoted, however well-meaning, however high-minded. If it be true, as has been said, and I believe it is, that a large part of our hospitals are filled with the errors of our educational system, it is the duty of a wise Government not only to remedy these errors, but also to see to it that while remedial educational measures are taken, medical assistance is rendered in the most efficient, thorough, and systematic way. This need not deter private effort and charity. As pointed out, and I repeat it by way of emphasis, there is one great blemish in all our charities. It is this overlapping. Overlapping means waste, and we ought all to do what we can to eradicate that weed that is impairing the usefulness and vitality of all charitable effort. I cannot but agree with the Minority Report of the Poor Law Commission when it says:—"It is impossible after considering all the evidence to avoid the conclusion that what is before all else needed with regard to curative treatment of the sick poor is the establishment of one united public medical service." In that connection let me say that it is a matter for congratulation that at last there seems a prospect that London will be provided with an efficient metropolitan ambulance service under municipal control, supplied with motor vehicles which can be summoned by telephone calls. The urgency and importance of this reform cannot be overrated.

### We Have to Go On.

But I am afraid I have strayed away from the subject-matter of my toast, and have kept you much too long. Let me come back to the Great Northern Central Hospital. This hospital does splendid work. It is a necessity. To many a poor sufferer it is almost providential. Let us remember that. We may never reach the time when our hospitals are empty, when sickness has disappeared from this earth, but we can and must reduce, and in many cases prevent, sickness and suffering. When I walked through these wards filled with so much suffering and sadness, but replete, too, with wonderful patience, courage, and kindness, I felt very strongly, as one always does afresh when brought face to face with suffering and sadness, that we must all stand together and fight poverty and sickness; not by spasmodic remedies, but by going to the root of things and asking ourselves and asking the Government of the day, Why are all these people ill and in pain? Is it all inevitable? Can nothing be done to prevent at least some of it in the future? For prevention is better than cure.

And let me here, in conclusion, quote a fine passage from one of Mr. Wells's books. Standing on Westminster Bridge at night with a friend, and looking at London, which he says was beautiful that night with her dark and meretricious splendour, he makes this reflection: "But it was clear to us the thing for us two to go upon was not the good of the present nor the evil, but the effort and the dream of the finer order, the fuller life, the banishment of suffering to come. 'We want all the beauty that is here,' said my friend, 'and more also. And none of these distresses. We are here—we know not whence nor why—to want that and to struggle to get it, you and I and ten thousand others, thinly hidden from us by these luminous darknesses. We work, we pass—whither I know not, but out of our knowing. But we work—we are spurred to work. That yonder—these people are the spur for us who cannot answer to any finer appeal. Each our measure must do. And our reward? Our reward is our faith. Here is my creed to-night. I believe out of me and the Good Will in me and my kind there comes a regenerate world, cleansed of suffering and sorrow. That is our purpose here—to forward that. It gives us work for all our lives. Why should we ask to know more? Our errors—our sins—to-night they seem to matter very little. If we stumble and roll in the mud, if we blunder against each other and hurt one another, we have to go on,' said my friend, after a pause." And so I say to you. We have to go on. God bless the Great Northern Central Hospital.

### SIR JOHN DICKSON POYNDER.

In rising to respond to the toast of the evening, said: I should like to voice what must be uppermost in the minds of everybody at this banquet, and express to our chairman our deep indebtedness to him for his generosity and kindness in coming here this evening and presiding over our gathering. We are also greatly indebted to him for the very instructive and interesting speech that he has delivered, and we are also grateful to him for the unremitting energy and interest that he has displayed towards our efforts in the Great Northern Central Hospital ever since the first day that he was good enough to accept the invitation up till to-night. He has left no stone unturned to make this banquet successful in its results. (Applause.) In Sir Edgar Speyer we have one of those gentlemen who has on many previous occasions been a ready and willing victim to that kind of philanthropic work, and to-night by presiding at our dinner he has added one more to those many occasions when he has pleaded the cause of charity. Perhaps I may be allowed in expressing what may be uppermost in the minds of all present, to offer to Sir Edgar Speyer our very warmest congratulations upon the high distinction that has recently been conferred upon him by his Majesty as a recognition of those many and varied services that he has rendered to the State. (Applause.)

### The Present Position.

We have had put before us this evening in very lucid terms by our chairman the exact position of our hospital, and looking back now for a number of years as chairman of the hospital, I must cordially confess that with all the different vicissitudes and ups and downs that I have seen the institution pass through in days gone by I have never felt that the immediate outlook presented so gloomy and critical a view as it does at the present moment. You have been told quite truly that we have been affording accommodation with our 179 beds, and the cost of it is something like £17,000, and we have this year and last year only been able to raise something less than £4,000 of that total. It is quite true that the accumulated debt owing to the bankers now amounts to something like £14,000. These are figures so formidable and so critical for the immediate future that we cannot blink our eyes to the fact that unless some very substantial support comes to our aid in the immediate future some very substantial change must take place in the procedure and method of administration in our institution. We have had this evening certain advice given to us as to methods by which we might exercise closer economy in the administration than has hitherto been the case. Speaking on behalf of my colleagues I am quite ready to admit that we are by no means a perfect board of administrators, and I have not the slightest doubt that by a scrutiny of our accounts certain defects could very readily and easily be found. I think probably on a similar close scrutiny being made defects could be found in nearly every hospital in London, but what I am prepared to say is that whatever our imperfections may be we do to the best of our ability effect every conceivable economy we can. It is quite true that our beds show a somewhat higher average than might be expected, but I must candidly admit that is due almost to one class of expenditure—namely, the surgical and medical department, and anyone who is conversant with hospital administration, as I am, will fully appreciate with me the difficulties of restraining the enthusiasm of those who preside over those particular departments.

### The Question of Reforms.

Whatever imperfections and whatever extravagancies may be shown in our hospital, I believe our imperfections, which are inherent from what is rapidly becoming an obsolete practice amongst all hospitals, and I would point this out, that it is an extremely difficult thing for an isolated single hospital to take upon itself to effect great changes and reforms. I know, and my colleagues know, many things that might be done which we would like to do, and willingly would do, but we have always before us this fact that we depend for our daily maintenance upon the generosity of the public, and that generosity is bound to a very large extent by prejudice, and were we an isolated hospital to go forward and effect some radical reform, I am very much afraid that before the radical change took place the prejudice might be so great amongst those who are in the habit of supporting us that we should run the very severe risk of finding ourselves in a state of extinguishment before the reform came about.

### Hospitals and the State.

Some outside enthusiasts tell us that the time has arrived when all hospitals should be put upon the rates, and should be administered by the local authority. Then we are told by others that the State should intervene and a and a State subsidy should be granted for the maintenance of the hospitals. There is more to be said for that, in my judgment, than there is for the former suggestion, but I would point out that both of these suggestions are fraught with great difficulties. There are many difficulties and disabilities which would have to be overcome before either of those proposals could be resorted to. What I would suggest myself, from a practical point of view, is that before any vital change was made in that direction there should be an exhaustive inquiry into the whole question. It is something like eighteen years ago since a public inquiry was made, appointed by the Government, into the question of the hospitals of London. It was in the year 1892 that the Committee considered and inquired into the whole question, presided over by Lord Sandhurst. At that date, the hospitals, as they are now, were in a critical condition, although I believe the position to-day is more serious. The findings of that Committee were, that if any reform is to take place in hospital treatment in London it must be under a system of co-ordination in hospitals. There must be appointed some central body in which the whole administration of hospitals should be co-ordinated so that you might have as a result some uniform system over the whole of the Metropolis, and the Report went on to say that unless something of that kind was done there could be no other suggestion than that of the hospitals falling eventually upon the rates or into the hands of the State.

### The King's Fund.

I am one of those who always welcomed the establishment of a central fund like the King's Fund over the hospitals of London. I think that very beneficial results have already been shown from the existence of that fund and its committees. We have done something at any rate, although it may be modest in its degree at present in attempting a uniform treatment in certain branches of our hospital administration, and there is also the great advantage with these central bodies that they act as a receptacle of a large and increasing fund drawn from many sources which is placed at the disposal of the different hospitals in London. It must always be remembered that this central receptacle for donations and bequests, falling in as they do from all directions, has to a certain extent, and in some cases to a rather serious extent, a corresponding detrimental effect upon the local subscriptions that come to hospitals. I believe the main function of this central fund is that it should do its best to maintain all those useful hospitals that are situated in the poorer districts of London.

### The Staff.

We have this evening made one other departure in cutting down to the most modest dimensions the toast list, but there is one toast which has been omitted to-night on account of this law of brevity which really should always be included in a banquet of this character, and as it is not, perhaps I may be allowed to say one word in respect to it. It has always been the custom to have proposed the toast of the medical staff of the hospital. There are no group of gentlemen who take part in the work of this institution who are more important to its success and usefulness than are the honorary medical staff. We have been in years past, and I am glad to say we are to-day, peculiarly fortunate in having the advantage of the services of many eminent surgeons and physicians drawn from different parts of London. They always have to come great distances to the hospital, and they have to deny themselves and to sacrifice many lucrative professional hours in order to give that assiduous and most beneficent service in the interests of this hospital which they do give.

### The Work of the Hospital.

The financial situation of the hospital is not the only serious thing that it may involve, though I hope it will not do—a diminution in the accommodation to those poor people who live in the borough of Islington and the neighbouring boroughs, because we do not confine our useful work merely to the borough of Islington. We have always admitted those who live in the adjoining parishes, but what I want to point out is that the financial position will not only diminish our usefulness in one direction, but this insufficiency of funds, if it is allowed to continue, will have a very detrimental effect upon the medical and surgical scientific development of the hospital. We hear a great deal in these days of the rivalry of armaments among the nations of the world, but I believe the nations that will stand pre-eminent in commerce and in intellect will be the nations that can show the highest standard of material and physical efficiency among its population.

On these two scores it is of paramount importance that we should use every possible effort we can to maintain our hospital in a high state of efficiency. I quite agree with what Sir Edgar Speyer says, that if we cannot do it on an economic basis, that we must set our house in order, but I cannot believe that in this great Metropolis of ours, with the enormous wealth that is to be found among thousands of the population, that such a calamity will be allowed as a diminution of the work of a hospital like this, which, in Islington with its population of 340,000, giving as it does only sufficient accommodation to those who are sick. I ask you to give, and to give as generously as you can, because in so doing I can answer for it you are giving to a good purpose—you are giving for the purpose of finding sufficient medical and surgical accommodation for the poor sick of North London who knock at the doors of the Great Northern Central Hospital, and who hitherto have always been admitted, and who, I hope, in the days to come will continue to have the doors kept wide open for them. (Applause.)

### THE STEWARDS' SUCCESSES.

List of Contributions through :—Right Hon. Sir Edgar Speyer, £1,692 2s. 6d.; the Worshipful Livery Companies, £354 2s.; Corporation of the City of London, £100; Sir John Dickson Poynder, Bart., M.P., £786 16s.; H. J. Tennant, Esq., M.P., £150; G. H. Alexander, Esq., £37 0s. 6d.; J. B. Batchelor, Esq., £6 16s.; Dr. E. Clifford Beale, £199 10s.; Mrs. Lansdowne Beale, £46 15s.; T. Fraser Black, Esq., J.P., £42; C. B. Brooke, Esq., £59 17s.; A. H. Cæsar, Esq., £42; T. C. Cartwright, Esq., £21 14s. 3d.; W. Curnick, Esq., £5 5s.; W. F. Dewey, Esq., £424 4s. 3d.; Oswald Fitch, Esq., £5 5s.; F. W. Fletcher, Esq., F.C.S., £23 2s.; F. Harper Greig, Esq., £10; Frederick Hammond, Esq., F.R.I.B.A., £33 5s. 9d.; Percival Hart, Esq., £91 8s.; Henry J. Hazel, Esq., £8 8s.; Fred. Hodge, Esq., £20; R. J. Bridgland, Esq., £31; Dr. Charles Ironside, £36 16s. 6d.; Dr. Robert Knox, £60 9s.; D. Marshall Lang, Esq., £7 5s.; Frank Lawson, Esq., £114 15s.; Dr. and Mrs. W. A. Malcolm, £32 4s. 6d.; Mrs. Everard Milman, £5 8s.; Dr. Alexander Morison, £526 5s.; A. Pinnock, Esq., £28 15s.; Charles Pinnock, Esq., £10 15s.; Rev. C. J. Procter, M.A., £31 10s.; Dr. C. A. Casterton-Smelt, £75; C. Walton Sawbridge, Esq., £69 3s. 6d.; J. L. Smithett, Esq., J.P., £312; David Waterlow, Esq., M.P., £36 0s. 6d.; Mrs. David Waterlow, £63 19s. 6d.; Mr. and Mrs. G. B. Mower White, £36 3s.; Councillor C. E. Shepherd, £6 6s.; H. E. Mathews, Esq., £24 3s.; James Blackney, Esq., £14 14s.; C. E. Bradbury, Esq., £11 11s.; V. Warren Low, Esq., F.R.C.S., £4 8s.; T. Pargeter, Esq., £4 4s.; Mrs. Vincent, £33 10s. 6d.; Sir Robert Burnet, £22 2s.; Alderman T. W. Saint, £9 19s. 6d.; J. Harry Auckland, Esq., £16 4s.; General list of donations per Secretary, £686 19s. 7d.—Total, £6471 2s. 4d.

